# Digital health and COVID-19: challenges of use and implementation in sub-Saharan Africa

**DOI:** 10.11604/pamj.2021.38.240.27948

**Published:** 2021-03-07

**Authors:** Yidnekachew Girma Mogessie, Blaise Ntacyabukura, Dawit Tesfagiorgis Mengesha, Mohamed Babiker Musa, Marie-Claire Wangari, Nsabimana Claude, Nit Buntongyi, Don Eliseo Lucero-Prisno

**Affiliations:** 1 St. Paul's Hospital Millennium Medical College, Addis Ababa, Ethiopia,; 2 Karolinska Institutet, Solna, Sweden,; 3 Faculty of Pharmacy, Omdurman Islamic University, Khartoum, Sudan,; 4 Nazareth Hospital, Nairobi, Kenya,; 5 Ministry of Health, Kibagabaga District Hospital, Kigali, Rwanda,; 6 Faculty of Medicine, University of Puthisastra, Phnom Penh, Cambodia,; 7 Department of Global Health and Development, London School of Hygiene and Tropical Medicine, London, United Kingdom,; 8 Faculty of Management and Development Studies, University of the Philippines (Open University), Los Baños, Laguna, Philippines

**Keywords:** Digital health, COVID-19, sub-Saharan Africa

## Abstract

COVID-19 is a global health emergency that exposed the gaps in health systems globally, especially in sub-Saharan Africa home to many fragile healthcare systems and a region beset with a large burden of disease. Various mitigation strategies have been put in place to stop the spread of COVID-19 and management of patients in sub-Saharan Africa. However, much still need to be done. Digital health provides the promise for the continent to bridge the gap in decreasing the negative impact of COVID-19 and effectively mitigate the pandemic. This commentary argues how countries in sub-Saharan Africa need to embrace the use of digital health in public health interventions to vigorously mitigate the COVID-19 pandemic and to contribute towards attaining universal health coverage (UHC).

## Commentary

The COVID-19 pandemic has been challenging and has been disrupting health systems worldwide; and sub-Saharan Africa has not been spared. Access to essential health interventions was already limited in sub-Saharan Africa prior to the COVID-19 pandemic [1]. The use of digital health to address this issue has been showing inroads in the health systems of the continent. Health technology startups have been initiated, though they were struggling to be mainstreamed and to overcome Africa´s fragile and unattractive healthcare system. Digital health has been employed during the pandemic and has been shown by several countries with digital infrastructures and innovation to boost outbreak communication, contact tracing, reinforce lockdowns, smoothen logistic flow and implement e-learning [2].

**COVID-19 information management:** during the pandemic, there was a rise in citizens´ inquiries about correct information on COVID-19. For example, the call center of the Nigeria Centre for Disease Control (NCDC) was bombarded with queries. NCDC applied artificial intelligence that can simultaneously communicate with thousands of callers [2]. Ethiopia has developed a data-driven health information portal with a rapid response component to citizens´ questions using call centers [3]. Rwanda used drones to promote health education to unreachable populations [4]. Sierra Leone, South Africa, Morocco and Tunisia imposed lockdowns and determined citizen´s compliance by issuing alerts and announcements using drones [5].

**Contact tracing, e-learning and logistics flow:** health systems in Africa are strained and have minimal capacity to address the pandemic. Digital infrastructure plays a substantial role in the containment of COVID-19, capacity building of the workforce and in smoothening flow of logistics [6]. Ethiopia has designed several apps that trace contact, share data and patient information among the health workers, reducing the delay in announcing test results and improving the logistics flow [7]. They also developed an algorithm that provides smooth access to e-learning for healthcare professionals all over the country [7]. Drones were used in Rwanda and Ghana to distribute personal protective equipment (PPE) and COVID-19 test kits [5]. In Rwanda, information is entered in the District Health Information Software2 (DHIS-2) system on the spot, allowing timely intervention in tracing, isolating and testing suspected cases [4].

**Data generation and patient care:** there is a significant lag in electronic medical record (EMR) adoption and adhering to traditional (paper-based) patient chart documentation in many African countries. Ghana, Kenya and Ethiopia have integrated COVID-19 signs and symptoms into existing EMR platforms while triaging and utilizing EMR to track the resolution of respiratory symptoms in COVID-19 centers [2,8,9]. Rwanda and Tunisia have also introduced robots to monitor patient vital signs, reducing contact with healthcare workers [4,5].

**Challenges:** several challenges in mainstreaming digital health in sub-Saharan Africa during the COVID-19 pandemic are still observed. Before the crisis, targeted users of health technology products in various parts of Africa were reluctant to integrate the innovations into the health-care system. This made social distancing rules and other infection prevention control protocols in Africa difficult to implement [2,9]. A big part of Africa needs access to the internet which creates a considerable obstacle in developing digital infrastructures and rejecting stakeholders investing in digital health [10]. Existing challenges such as lack of financial incentives and priorities, inadequate electricity supply and connectivity and lack of well-trained workforce were the main obstacles to implementing digital health during the pandemic [2,9].

**Recommendation and conclusion:** digital health has a tremendous role in revolutionizing the traditional health system in sub-Saharan Africa. Various developed nations effectively utilized digital health to minimize the impact of COVID-19, from the identification of the first case to outbreak containment to recovery and resumption of post-COVID-19 living. To implement effective digital health, challenges related to affordability, infrastructure and human resource capability, must be addressed. Therefore, political commitment to sustainable funding, collaboration, cooperation among stakeholders and workforce training remains crucial in anchoring the COVID-19 response in sub-Saharan Africa.
